# The Maximum Bite Force for Treatment Evaluation in Severely Affected Adult SMA Patients—Protocol for a Longitudinal Study

**DOI:** 10.3389/fneur.2020.00139

**Published:** 2020-02-25

**Authors:** Teresa Kruse, Helmar C. Lehmann, Bert Braumann, Gereon R. Fink, Gilbert Wunderlich

**Affiliations:** ^1^Poliklinik für Kieferorthopädie, Universität zu Köln, Medizinische Fakultät und Universitätsklinikum Köln, Cologne, Germany; ^2^Zentrum für Seltene Erkrankungen, Universität zu Köln, Medizinische Fakultät und Universitätsklinikum Köln, Cologne, Germany; ^3^Klinik und Poliklinik für Neurologie, Universität zu Köln, Medizinische Fakultät und Universitätsklinikum Köln, Cologne, Germany

**Keywords:** spinal muscular atrophy, motor neurons, antisense oligonucleotides, bulbar neuromuscular function, masticatory force, piezoelectric transducer

## Abstract

Spinal muscular atrophy (SMA) is a severe neuromuscular disorder characterized by the degeneration of motor neurons in the spinal cord, and comprises a broad clinical spectrum. With the advent of new therapies (e.g., Nusinersen) for patients of all ages and disease stages, sensitive clinical measures are needed to detect slight changes in muscle force even in immobilized, severely affected patients often unable to move limbs. As for these patients, well-established outcome scales set out to evaluate motor function do not work properly, we propose measurement of maximum bite force which is able to detect subtle changes of bulbar function. Requirements for this approach are mentioned, challenges are discussed, and first insights from a pilot study are presented. Finally, a study design is proposed to evaluate the measurement of maximum bite force during the follow up of SMA patients with and without a disease modifying therapy.

## Introduction

Spinal muscular atrophy (SMA) is a severe neuromuscular disorder characterized by the degeneration of motor neurons in the spinal cord resulting in muscular atrophy, weakness and paralysis (1). The disease presents with a diverse range of phenotypes of motor impairment and comorbidities with onset and severity of disease providing the basis for the classification of the different subtypes of SMA: very weak infants unable to sit unsupported (type 1), non-ambulant patients able to sit independently (type 2), ambulant patients with childhood (type 3) and adult onset SMA (type 4). The era of restriction to supportive therapies ended when 2017 Nusinersen was approved for all patients with SMA. Interestingly, approval was based on studies (ENDEAR and CHERISH) in children with SMA type 1 and 2 (2, 3). Additional open-label studies (CS1, CS2/CS12) confirmed the results. These are the only studies that included older patients (five patients were 15–16 years old suffering from SMA between 9 and 15 years) (4). Therefore, treatment of adult SMA patients faces a lack of clinical data concerning efficacy and safety of treatment beyond childhood and long disease duration. Furthermore, concerning disease severity and age many SMA patients are far away from the existing study populations (5). Finally, severely affected patients are immobilized, unable to walk, stand or sit, and often are even unable to move limbs. For these patients, well-established outcome scales set out to evaluate motor function do not work properly. In these individuals, the 6-Minute-Walk-Test (6MWT) is no longer applicable, and descriptive scores like the Hammersmith Functional Motor Scale Expanded (HFMSE), Revised Upper Limb Module (RULM) or Children's Hospital of Philadelphia Infant Test of Neuromuscular Development (CHOP INTEND) reach their limits due to immobility of the patients. HFMSE and RULM are insensitive to detect marginal changes in neuromuscular function, as they evaluate gross motor skills only (6). While CHOP INTEND is a more sensitive scale, it is tailored to assess motor function in infants and has not been validated for all types of SMA (7, 8).

This lack of objective measures is further supported by preliminary data from a German cohort of adult SMA patients treated with Nusinersen since its approval in 2017. Whereas in “walkers,” clinical improvement was objectified by marked changes in the above mentioned scores HFMSE and RULM remained largely unchanged in immobilized “sitters” and “non-sitters,” although patients reported clear improvement in muscle function during therapy (personal observations, unpublished data).

To evaluate motor function and thus drug efficacy in patients of all ages and types of SMA more sensitive clinical tools are needed urgently.

Still feasible in patients at an advanced stage of SMA is the evaluation of bulbar functions. Degeneration of alpha motor neurons leads to progressive muscle weakness involving spinal muscles earlier more severe than craniofacial and bulbar muscles (9, 10). Although functional abilities as speaking, chewing, and swallowing are affected at late onset (11), they are currently not evaluated in routine diagnostic procedures, given that scales like the HFMSE, RULM and CHOP INTEND do not take into account impaired bulbar function. To date, bulbar function in SMA patients has only been assessed for descriptive purposes, based on the subjective assessment of clinical findings or on individual in-house standard scales like the “Swallowing Impairment Scale” by Prosiegel or the “Bogenhausen Dysphagia Score” (BODS-2) (12, 13). Extending the set of existing motor scales with a more reliable outcome measurement of bulbar function seems highly desirable, especially in case of severely affected patients (14). A promising option in this regard is the measurement of maximum bite force, an established method to quantify muscle strength in the stomatognathic system.

## The Measurement of Maximum Bite Force

Maximum bite force is an indicator of the functional state of the masticatory system (15). Sensitive to nuanced changes of muscle force, it may be useful to assess changes in the bulbar function due to progressive neuromuscular degeneration or treatment-related effects.

Maximum bite force in humans is generally determined by the combined action of the jaw elevator muscles modified by craniomandibular biomechanics and reflex mechanisms (16). Its empirical assessment is frequently used in dentistry, e.g., to evaluate therapeutic effects of prosthetic devices. Reported values of maximum bite force in healthy patients vary greatly (17, 18) due to physiological as well as methodological factors that may induce variation: Various methods and recording devices exist, ranging from simple springs to more complex electronic devices; most appliances achieve an accuracy of +/− 10 N or a precision of 80% (19–21). The choice of the most adequate recording device, its setup, and the measurement protocol depends on the specific research question and on the population to be examined. General physiological and anatomical characteristics of the subjects must be taken into account as influencing factors, such as craniofacial morphology, age, gender, periodontal support of teeth, or dental status (16). Usually, they cannot be eliminated by the researcher but can be held constant as they rarely change during measurement. Potential cost-efficient alternatives to bite force measurements, for example the evaluation of masticatory performance using color-changeable chewing gum (15), are instead much more sensitive to these influencing biases. Less tangible physiological factors influencing bite force measurement are patients' motivation, their current condition, or a potential learning effect (22). In case of a disease affecting neuromuscular function, an alteration of maximum bite force has been shown previously (10).

## Bite Force Measurement in SMA Patients: Previous Research

Early approaches to measure bite force in SMA patients date back to 1999: Granger and colleagues showed that absolute levels of maximum bite force in SMA patients were reduced to 50% compared to a healthy control group (10). More recently, van Bruggen et al. performed tests in patients with SMA type 2 and 3 providing evidence that mandibular dysfunction in SMA reflects bulbar involvement. Maximum bite force was 19% lower in SMA patients than in control subjects. Especially in patients with SMA type 2, bite force, mandibular range of motion, active maximum mouth opening and the number of functional tooth units was reduced (9). To date, bulbar function in SMA patients has only been assessed cross-sectionally. Newly introduced causal treatments of SMA, however, call for a longitudinal measurement of changes in bulbar function over time.

## Bite Force Measurement in SMA Patients: Challenges

For the assessment of maximum bite force in SMA patients, acquiring *reliable measures over a longer time period* represents a first challenge. Focusing on intra- instead of inter-individual variation, bite force measurements over time should be free of time-varying confounding. Unlike most dental research, aiming at an exact representation of absolute levels of bite force, longitudinal assessments of changes in bite force call for reliable and not necessarily valid measurements. Systematic measurement error is irrelevant as long as it remains unchanged over time. Moreover, mechanical and methodological influences need not be fully eliminated but should simply be held constant, such as patients' head position (23) or the measurement position in the dental arch (24). Physiological characteristics of the patients, like SMA type, age, sex, anatomic conditions, remain mostly stable during the measurement period. They can either be neglected, controlled for in the statistical analysis or be used for subgroup analyses. Another, less tangible confounder is a potential learning effect: over time, patients get accustomed to the measurement, which may lead to increased values in measured bite force (22). To avoid such learning effects, patients should get familiarized with the procedure prior to the actual measurement period.

A second challenge to be met are SMA patients' special *anatomical conditions which may impede bite force measurement:* Bite force is severely reduced in SMA patients and should not be further inhibited by measurement appliances. Maximum bite force decreases with increasing interocclusal distance (25). Accordingly, thin intraoral sensors should be used—a *conditio sine qua non* in light of patients' limited mouth opening. A lack of occlusal contact or of periodontal support in SMA patients may lead to reduced values in measured bite force, due to the general limitation by periodontal or temporomandibular joint receptors (26, 27). To increase the area under load, bilateral measurements may be helpful. In addition, the sensor's surface may evoke a subconscious inhibition of maximum adduction: here, a slightly soft surface is advantageous (28).

## Toward a Customized Device for SMA Patients: A Pilot Study

The development of the protocol relied heavily on experiences gained in a small pilot study including two female adults with infantile onset SMA, later classified as SMA type 2 in view of their long-term survival, during their first year of nusinersen therapy (paper currently under review). Patients' motor abilities were limited to minimal movements of single fingers, a reduced facial mimic and reduced chewing and swallowing. Missing teeth, a severe class II malocclusion, and bilateral temporomandibular contractures with limitation of mouth opening had to be considered in the design of the bite force measurement device.

**Figure 1:**
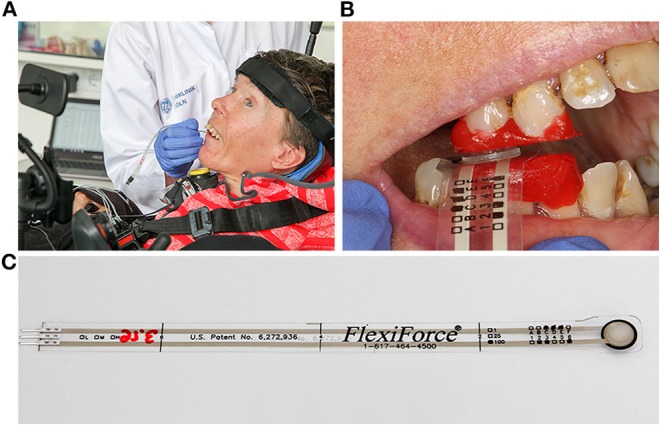
Customized device for bite force measurement in SMA patients. **(A)** Use of a thin piezoelectric force sensor on a severely affected adult patient with SMA type 2. **(B)** Sensor placed unilaterally in a region of good intercuspidation. Teeth are partially covered by occlusal bite blocks protecting the sensor from damage, assuring good contact, and precise repositioning. **(C)** The thin Flexiforce transducer A201-100 (Tekscan Inc., USA) guarantees minimal jaw separation. Written informed consent was obtained from the individual for publication of this image.

The use of a thin piezoelectric force sensor, the Flexiforce A201 (Tekscan, Boston, MA, USA), guaranteed minimal jaw separation (Figure 1): Featuring a load range of 440 N and a sensitivity of 0.01 V/N and a thickness of 0.2 mm, it can serve as a validated, low cost device to measure bite force in SMA patients in a clinical setting. Forces exerted on its sensing area, 1 cm diameter in size, cause a change of the sensor's conductance, generating an electric current which can be amplified with a digital recorder (21, 29). The reliability of piezoelectric sensors is given as 93% (30). To achieve such high accuracy and reliability with piezoelectric sensors, equilibration and calibration of the sensor is essential (31).

The sensor was placed unilaterally in a region of good intercuspidation of the premolars or molars. Given the importance of a precise repositioning of the sensor in each measurement (24) and of a good contact to the sensor, teeth were partially covered by intraorally fabricated occlusal bite blocks with a soft surface, which at the same time protected the sensor from damage by the occlusal relief (Figure 1B).

Bite force was measured repeatedly for 1 year, before, during and after the application of Nusinersen. Initial maximum bite forces of the two surveyed adult patients with SMA type 2 was substantially lower than normal (35.7 N and 63.4 N compared to values at ~300 N in a healthy female adult). HFMSE and CHOP INTEND remained nearly unchanged at a low level over the entire year of observation. The results of 550 bites, clustered in 55 measurements points, indicated systematic and statistically significant changes of maximum bite force that coincided with medication. Over the course of four loading doses of nusinersen, bite force levels reached a significantly higher level compared to the initial state in both patients (*p* < 0.05). During the subsequent periods without drug administrations, maximum bite force decreased. It could be speculated that maximum bite force changes with the application of nusinersen in adult SMA patients, potentially indicating an altered bulbar function.

## Integrating Bite Force Measurement in SMA Patients' Clinical Routine

To establish bite force measurement in the routine clinical examination of SMA patients, further investigations are necessary. In addition, more readily available appliances seem desirable, guaranteeing equally reliable measurements while rendering professional dental support unnecessary.

**Figure 2:**
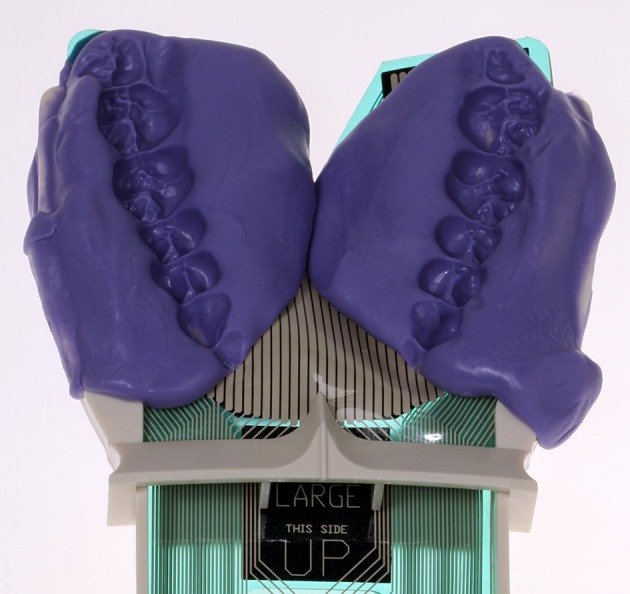
Individually adjusted piezoelectric T-Scan sensor foil (Tekscan Inc., USA) with individually adjusted surface (dental silicone, Honigum, DMG, Hamburg) to compensate for dental or skelettal anomalies and to ensure a reproducible position in each measurement.

The recently introduced combination of a larger piezoelectric intraoral sensor and a new I-Scan software (Tekscan, Inc., South Boston, MA) provides a central advantage: covering the entire dental arch, it greatly simplifies reliable repositioning without the need of customized bite blocks. Prior to the first measurement, the surface of the sensor is adjusted to each patient's dental arch using dental silicone (Honigum, DMG, Hamburg). Doing so helps to compensate for some of SMA typical dental or skeletal anomalies by flattening the contact surface (Figure 2). Compatibility of the intraoral T-Scan sensor with the I-Scan software guarantees an accurate calibration, equilibration, and quantification of bite force (31). The combination of T-Scan sensor foil and I-Scan software has been successfully applied in preliminary studies (32, 33). Though less cost-efficient than unilateral bite force measurement via the Flexiforce sensor (29), this safe, straightforward clinical application may be an attractive alternative in the case of larger patient groups.

## Study Design for a Validation of the Approach

Bite force measurement needs to be applied in SMA patients of different disease stages and should be compared with established scales as CHOP INTEND, HFMSE or RULM. Measurement of intra-individual changes of maximum bite force over time in treated and untreated patients will contribute to the knowledge of degenerating bulbar function and treatment-related effects. In patients with severely reduced motor abilities, evidence of even marginal changes could be helpful, as other scores mostly fail to detect them.

To address these issues, the aim of this larger prospective observational, controlled, non-interventional, non-randomized, multicenter study is to evaluate oral function in patients with SMA of different types and severity over a longer time period. Patients with genetically confirmed 5q-associated SMA types 2 and 3 will be eligible to participate. To guarantee the feasibility of the measurements of bite force and tongue pressure, exclusion criteria comprise insufficient dentition in the posterior region, absent masticatory function, permanent ventilator support, and anchyloglossia. Patients will be allocated to two groups. The first group will contain patients supposed to be treated with nusinersen, and the second group those without nusinersen treatment. Changes over time will be compared between both groups. Furthermore, group 1 allows us to compare oral function before and after first application of nusinersen within each patient. A sample size of 20 patients per group is intended and seems realistic in the light of restricted enrollment of eligible patients.

The primary objective will be to measure maximum bite force and bite force fatigue over a time period of 22 months in nusinersen treated and untreated SMA patients. In order to provide a better overview of the overall oral function, several additional objectives will be pursued, including the measurement of tongue pressure and fatigue, maximum mouth opening, and swallowing impairment based on the Dysphagia Score by Bogenhausen (34, 35).

To account for natural variation in maximum bite force and tongue pressure over time, measurements in the untreated control group and measurements in healthy subjects will be mandatory. Tests for intra- and interrater variability will be carried out on healthy subjects prior the first measurement in SMA patients. For better comparison, the intervals of measurements have to be identical in each group. During each measurement, patients and controls will be asked to bite three times with maximum force for a duration of 3 s, with pauses of at least 30 s to avoid muscle fatigue. The same protocol will be applied for the measurement of maximum tongue pressure. Patients will be asked to press the tongue against an air-filled bulb (IOPI Medical LLC, Carnation, WA: Iowa Oral Performance Instrument) three times with maximum force for a duration of 3–4 s, with pauses of at least 30 s. This method is deemed as an effective standard to evaluate muscle strength of the tongue (36–38). For maximum bite force and maximum tongue pressure, values of these three measurements will be statistically assessed relying on the following descriptive statistics: arithmetic mean and standard deviation, median, 1st and 3rd quartiles as well as minima and maxima. Inferential statistics will rely on changes over time across the different groups, and linear mixed effects models will be used.

Given the evidence for faster fatigue among SMA patients (10), muscle endurance as an additional outcome measure is included. Patients and controls are asked to hold adduction at 60% of the previously determined maximum bite force and tongue pressure level as long as they can. Thereby, they will be able to visually monitor their force level. The time period until bite force or tongue pressure drops below 30% of the previously determined maximum force serves as an indicator for muscular fatigue. A comparison with results of routinely assessed clinical outcome scales HFMSE, RULM, ALSFRS, and 6-MWB will be done to reveal the diagnostic potential of the measurements of oral function in SMA patients. Variation over time of each measure will be analyzed using linear mixed models. All evaluations will be exploratory, no formal hypotheses will be tested.

## Conclusion

Quantifying changes of maximum bite force and oral function more generally is a promising approach to evaluate representative parts of bulbar function among SMA patients. Especially in patients with limited or without walking ability, further data in addition to established motor scales can be obtained, and potential functional changes due to treatment or due to further degeneration may be identified. The aim of this study is to validate the diagnostic potential of measurements of oral function in patients with SMA and determine its appropriateness for different subtypes.

## Data Availability Statement

The datasets generated for this study are available on request to the corresponding author.

## Ethics Statement

The studies involving human participants were reviewed and approved by Ethikkommission der Medizinischen Fakultät der Universität zu Köln. The patients/participants provided their written informed consent to participate in this study. Written informed consent was obtained from the individual(s) for the publication of any potentially identifiable images or data included in this article.

## Author Contributions

TK, BB, and GW contributed conception and design of the study. TK and GW wrote the first draft of the manuscript. All authors contributed to manuscript revision, read, and approved the submitted version.

### Conflict of Interest

The authors declare that the research was conducted in the absence of any commercial or financial relationships that could be construed as a potential conflict of interest.
